# Structural and functional studies of Spr1654: an essential aminotransferase in teichoic acid biosynthesis in *Streptococcus pneumoniae*

**DOI:** 10.1098/rsob.170248

**Published:** 2018-04-18

**Authors:** Xiao Han, Renhua Sun, Tatyana Sandalova, Adnane Achour

**Affiliations:** 1 Science for Life Laboratory, Department of Medicine Solna, Karolinska Institute, Solna, 17176 Stockholm, Sweden; 2 Division of Infectious Diseases, Karolinska University Hospital, Solna, 17176 Stockholm, Sweden

**Keywords:** teichoic acids, aminotransferase Spr1654, PLP, l-glutamate, crystal structure, substrate specificity

## Abstract

Spr1654 from *Streptococcus pneumoniae* plays a key role in the production of unusual sugars, presumably functioning as a pyridoxal-5′-phosphate (PLP)-dependent aminotransferase. Spr1654 was predicted to catalyse the transferring of amino group to form the amino sugar 2-acetamido-4-amino-2, 4, 6-trideoxygalactose moiety (AATGal), representing a crucial step in biosynthesis of teichoic acids in *S. pneumoniae*. We have determined the crystal structures of the apo-, PLP- and PMP-bound forms of Spr1654. Spr1654 forms a homodimer, in which each monomer contains one active site. Using spectrophotometry and based on absorbance profiles of PLP- and PMP-formed enzymes, our results indicate that l-glutamate is most likely the preferred amino donor. Structural superposition of the crystal structures of Spr1654 on previously determined structures of other sugar aminotransferases in complex with glutamate and/or UDP-activated sugar allowed us to identify key Spr1654 residues for ligand binding and catalysis. The crystal structures of Spr1654 and in complex with PLP and PMP can direct the future rational design of novel therapeutic compounds against *S. pneumoniae*.

## 1. Introduction

*Streptococcus pneumoniae* (*S. pneumoniae*) is the most frequently detected pathogen in community-acquired pneumonia and lower respiratory tract infections, resulting in very high morbidity and mortality rates in the world [1,2]. With the severely increasing number of antibiotic-resistant strains, the bacteria cell wall has attracted considerable attention due to its potential as a vaccine target. Teichoic acids (TAs) are cell wall polymers that contribute to up to half of the cell walls dry weight and play a fundamental role in Gram-positive bacteria physiology and pathogenesis [3]. In addition to binding cell surface proteins, TAs regulate the activity of cell wall hydrolases, cell wall elongation and cell division, as well as resistance to antimicrobial peptides and interactions with host factors [3–5]. This renders the TA biosynthetic pathway a possible and interesting target for therapeutic intervention, in our opinion. However, the molecular details underlying most of the molecular processes and steps for the bacterial production of TAs remain poorly understood [6].

**Figure 1.**
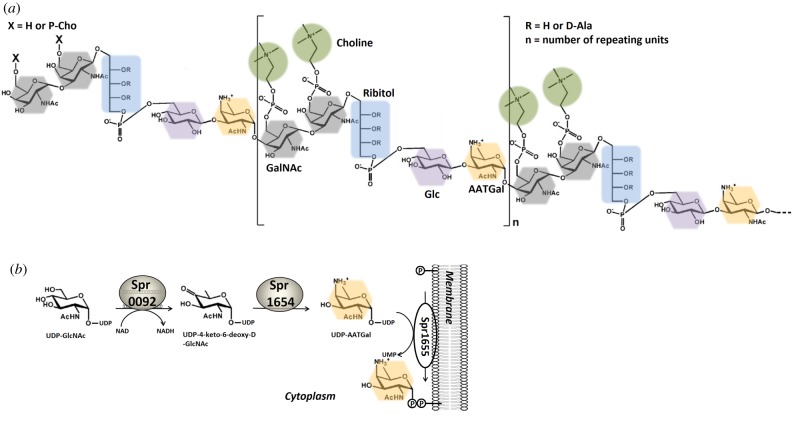
Structure composition of TA in *S. pneumoniae.* (*a*) Structural model of the pneumococcal TA. Each TA repeating unit (RU), which is unique for *S. pneumoniae*, contains 2-acetamido-4-amino-2,4,6-trideoxygalactose (AATGal), glucose (Glc), ribitol phosphate (Rib-P) and two *N*-acetylgalactosamine (GalNAc) residues carrying a phosphorylcholine (P-Cho) moiety. (*b*) Scheme of the three-step reactions necessary for the production of AATGal prior to incorporation in TA.

Most Gram-positive bacteria produce two distinct types of TAs: the wall TAs (WTAs) that are attached to the peptidoglycan (PG) and lipoteichoic acids (LTAs) that link to the membrane through a glycolipid anchor [6,7]. In contrast to most Gram-positive bacteria, the *S. pneumoniae*-associated WTA and LTA chains comprise identical and among all known bacteria unique repeating unit structures and length distribution [5,8,9]. Indeed, mass spectrometry and NMR spectroscopy analyses of LTA indicate that the repeating units of *S. pneumoniae* have an unusually complex structure, which begins with the rare amino sugar 2-acetamido-4-amino-2,4,6-trideoxygalactose (AATGal) [10–12] and contains glucose (Glc), ribitol phosphate (Rib-P) and two *N*-acetylgalactosamine (GalNAc) carrying phosphorylcholine (P-Cho) moiety on each residue [9,13] (figure 1*a*). It should be noted that TA biosynthesis in *S. pneumoniae* requires at least 19 cytoplasmic and membrane-associated protein-mediated steps [2,5] for the formation of precursors common to WTA and LTA, as well as their polymerization, transport through the cytoplasmic membrane and anchor to either the PG or the membrane [5,14,15]. Most of the hitherto identified essential genes involved in this process have been assigned to a functional category based on sequence homology or, much more rarely, experimental studies. However, the functions of several of the identified proteins remain hypothetical.

As assessed by mass spectrometry, the repeating units of the pneumococcal LTA begin with the rare deoxy sugar AATGal [11]. Practically all bacteria use deoxy sugars to decorate their surface, where these molecules participate in cell–cell interactions and toxin binding and/or serve as anchors for a large array of different surface bacterial proteins [16]. While bacterial carbohydrates are highly diverse, prokaryotes use approximately 100 different kinds of sugars and eukaryotes use less than 10 monosaccharides [17]. Most of the bacterial carbohydrate biosynthesis pathways share common intermediates that are synthesized in enzymatic reactions catalysed by homologous proteins.

*Streptococcus pneumoniae* must have a specific aminotransferase that transfers an amino group from an amino acid donor to the UDP-activated keto sugar in order to produce the amino sugar AATGal. Based on the presence in *Shigella sonnei* of an enzyme homologue to the genes encoding for spr1654, responsible for the synthesis of UDP-AATGal (UDP-2-acetamido-4-amino-2,4,6-trideoxygalactose), it has been suggested that Spr1654 in *S. pneumoniae* should play the same role [5,18]. Indeed, Spr1654 was suggested to catalyse the PLP-dependent addition of the amino group to the C-4 atom of the UDP-4-keto-6-deoxy-d-GlcNAc in order to form UDP-AATGal [11,18] (figure 1*b*). As UDP-AATGal is the substrate for the first membrane step of the TA synthesis pathway and becomes the initial sugar for the whole repeating unit in both LTAs and WTAs, Spr1654 therefore plays an essential role in this process. Indeed, following genetic disruption of Spr1654 (equivalent to SP1837 in *S. pneumoniae* TIGR4) in streptococcal cells, no colonies are formed on selective agar plates, demonstrating that Spr1654 is an essential gene for *S. pneumoniae* survival [19]. This makes Spr1654 an excellent target for the development of selective inhibitors that could abolish bacterial virulence by interfering with the biosynthesis of the cell wall. Such inhibitors could be particularly useful in the treatment of pneumococcal infections.

Here, we have determined the crystal structures of the apo-, PLP- and PMP-bound forms of Spr1654 at 1.9 Å, 2.2 Å and 2.4 Å, respectively. The three-dimensional structures revealed that Spr1654 has a fold characteristic of the type I aminotransferase family. Our structural and biochemical analyses further demonstrated the aminotransferase activity of Spr1654. The results of our spectrophotometry assays also revealed that l-glutamate is the optimal amino donor for Spr1654.

## 2. Results and discussion

### 2.1. Spr1654 is a PLP-dependent enzyme

**Figure 2.**
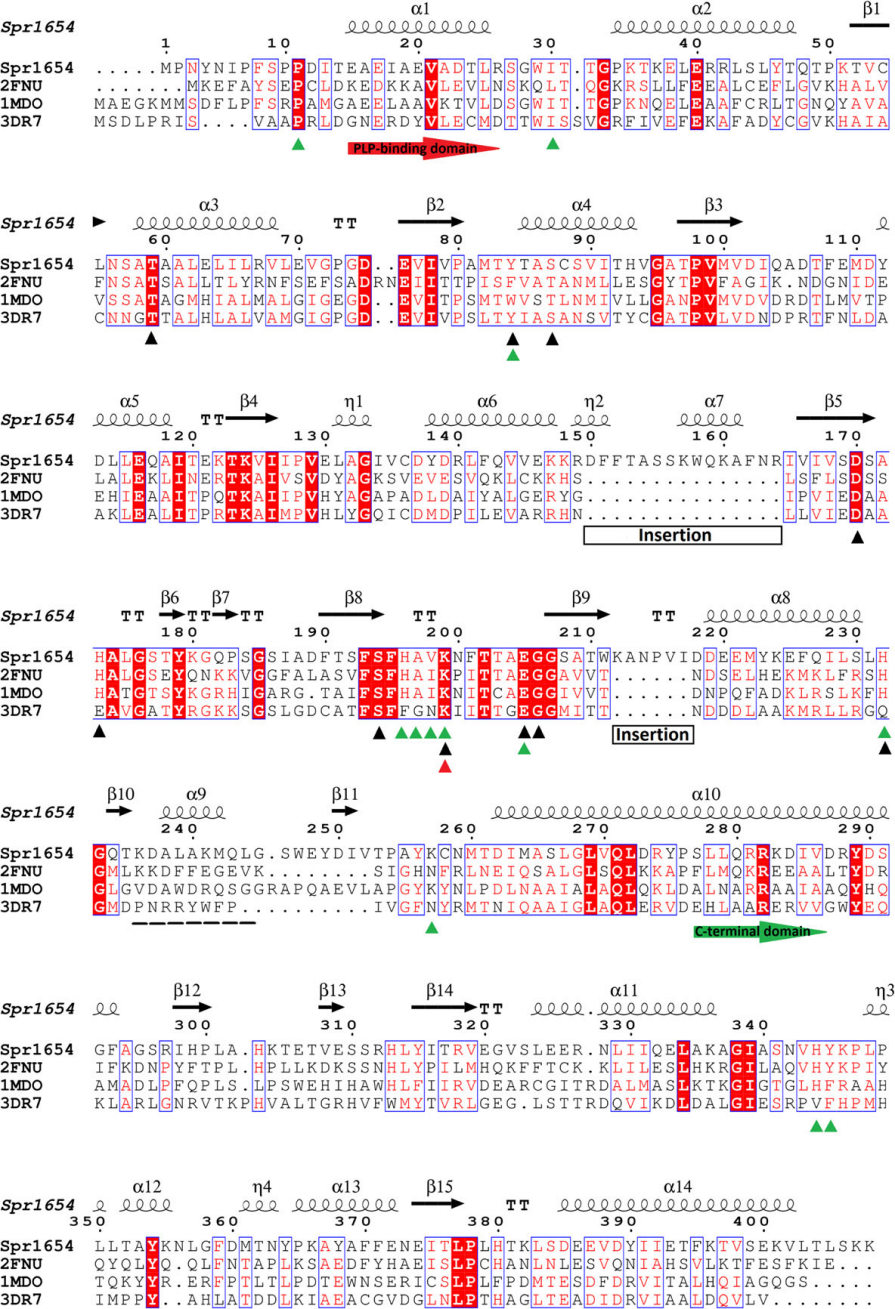
Sequence alignment of the streptococcal Spr1654 with PseC from *H. pylori* (PDB code: 2FNU), ArnB from *S. typhimurium* (PDB code: 1MDO) and GDP-perosamine synthase from *C. crescentus* (PDB code: 3DR7). Strictly conserved residues are boxed in red. Black and green triangles indicate residues involved in PLP and substrate binding, respectively. The catalytic lysine residue is indicated by a red triangle. Red and green arrows indicate the starting point of the large PLP-binding domain and the smaller C-terminal domain, respectively. The disordered region is marked with a dashed line. Two unique insertions of Spr1654 are indicated.

We initiated this study by running a BLAST search for the Spr1654 sequence against the Protein Data Bank (PDB) in order to identify possible functional homologues. The BLAST search revealed several sugar aminotransferases (SATs) from different bacteria as homologues to Spr1654. Three proteins from Gram-negative bacteria displayed the highest scores, including UDP-4-amino-4-deoxy-l-arabinose aminotransferase ArnB from *Salmonella typhimurium* (which catalyses the modification of LPS [20]), GDP-perosamine synthase from *Caulobacter crescentus* (which converts GDP-4-keto-6-deoxymannose to GDP-perosamine (GDP-4-amino-4,6-dideoxy-d-mannose) [21]) and PseC from *Helicobacter pylori* (which modifies sugars for the glycosylation of flagella [22]) (figure 2). Despite low overall sequence identity (29–32% identical residues) with these aminotransferases, residues known to be involved in cofactor and substrate binding, including the catalytic lysine (Lys199 in Spr1654) and the aspartate Asp170 (also in Spr1654), both necessary for PLP activation, are conserved (figure 2). Thus, these results strongly suggest that Spr1654 could be a sugar aminotransferase.

The full-length protein encoded by the *S. pneumoniae* gene spr1654 was overexpressed in *Escherichia coli* with a C-terminal his-tag, and the Spr1654-his was purified as described in the Material and methods section (electronic supplementary material, figure S1a). To assess if Spr1654 could use PLP for its reaction, we profiled the spectra of Spr1654 with and without PLP using absorption spectroscopy. The absorption spectrum of Spr1654, without the addition of PLP, displays the usual band at 280 nm. However, incubation of the protein with PLP results in the appearance of a second absorption peak at 420 nm, typical for the protonated form of the internal aldimine in PLP-containing proteins. We also observed an additional shoulder at 340 nm that can be attributed to deprotonated internal aldimine (electronic supplementary material, figure S1b), which is different from the absorption peak at 388 nm of free PLP [23,24]. Altogether, these results indicate that Spr1654 is a PLP-dependent enzyme.

### 2.2. The crystal structure of Spr1654 reveals a type I aminotransferase fold

The crystal structure of Spr1654 was determined in the apo-, PLP- and PMP-bound states at 1.9 Å, 2.2 Å and 2.4 Å resolutions, respectively (table 1). In all crystal structures, the electron density is well defined for residues Tyr4 to Thr404 within all the four subunits present in the asymmetric unit, except for a short helix comprising residues Ala238 to Leu244 that is missing due to flexibility in one subunit within each dimer (subunits B and D). This region of the protein could be part of the substrate-binding cleft, which explains its high mobility in the absence of substrate (see below).

**Table 1.** X-ray data collection and refinement statistics.

parameter	apo-Spr1654	Spr1654/PLP	Spr1654/PMP
data collection statistics			
resolution (Å) (range)	30.0–1.9 (2.0–1.9)	29.7–2.2 (2.3–2.2)	29.8–2.4 (2.5–2.4)
space group	*P*1211	*P*1211	*P*1211
cell dimensions for *a*, *b*, *c* (Å)	79.3, 100.0, 110.4	79.0, 99.3, 107.6	78.9, 99.6, 108.1
*α*, *β*, *γ* (°)	90.0, 97.7, 90.0	90.0, 97.7, 90.0	90.0, 97.5, 90.0
no. reflections^a^			
observed	348 605 (35 719)	169 716 (16 002)	213 232 (19 470)
unique	130 980 (13 145)	79 389 (7743)	64 471 (6344)
mean *I*/*σ*(*I*)	10.9 (2.3)	6.4 (2.0)	7.4 (2.0)
completeness (%)	97	95	99
*R*_merge_ (%)	6.3 (44.4)	9.8 (50.1)	15.1 (61.6)
*B*-factor from Wilson plot (Å^2^)	23.5	21.8	21.3
refinement statistics^b^			
*R*_cryst_ (%)	23.6 (33.4)	23.8 (32.6)	24.2 (31.4)
*R*_free_ (%)	26.5 (37.1)	28.8 (35.9)	28.5 (34.1)
no. non-hydrogen atoms			
total	13 277	13 383	12 804
protein	12 564	12 506	12 533
ligands	None	60	64
water	713	817	207
average *B*-factor values			
total	28.3	23.6	25.4
protein (Å^2^)	28.2	23.7	25.6
ligands (Å^2^)	None	24.9	25.3
solvent (A^°2^)	29.8	18.9	15.6
RMSD from ideal geometry			
bond length (Å)	0.014	0.012	0.011
bond angle (°)	1.6	1.53	1.52
Ramachandran plot			
% of residues in preferred regions	100	100	100
% of residues in disallowed regions	0	0	0

^a^Values in parentheses correspond to the highest-resolution shell.

^b^Five per cent of reflections were used for monitoring the refinement.

All known aminotransferases are dimeric [25]. Similarly, Spr1654 forms a homodimer in solution as defined by the size-exclusion chromatography elution profile. Two dimers, composed of subunits AB and CD, are present in the asymmetric unit of all the obtained crystals of all forms of Spr1654, with a buried surface area of 2490 Å^2^ per monomer (15% of the total solvent accessible surface area). The buried surface area between the two homodimers is less than 500 Å^2^, indicating that this is a contact resulting from crystal packing. The two dimers AB and CD are highly similar, with a root-mean-square deviation (rmsd) of 0.44 Å following the superposition of 780 Cα atoms. From now on, we will provide all the structural descriptions based on the AB homodimer. Also in order to distinguish residues from the two subunits within each biological dimer that participate in ligand binding, we always mark residues from the opposite subunit with an asterisk.

**Figure 3.**
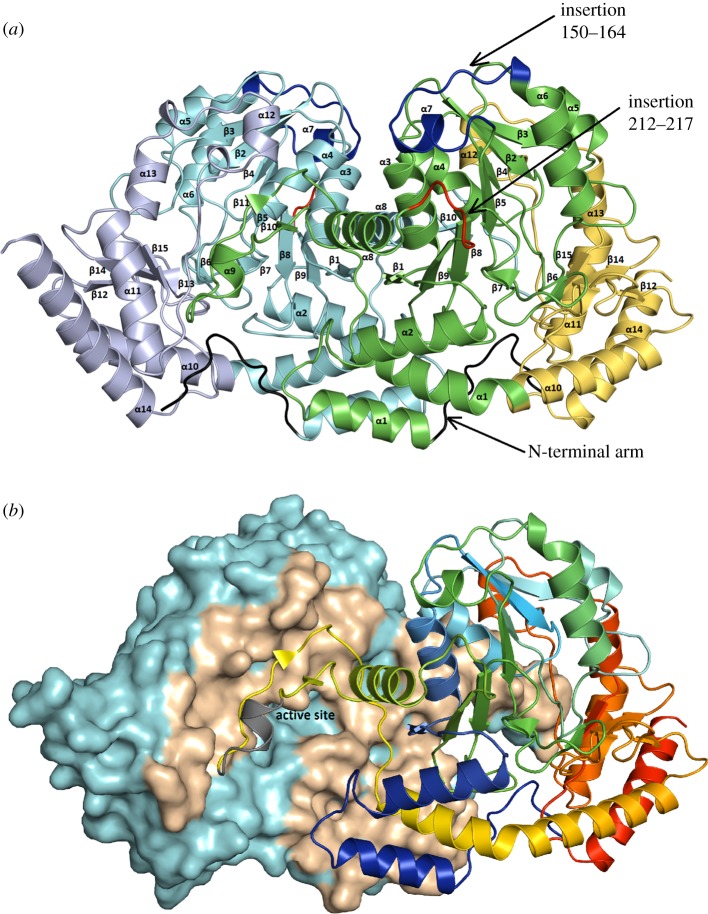
Architecture of the *S. pneumoniae*-associated Spr1654 aminotransferase. (*a*) The crystal structure of the Spr1654 dimer reveals a type I aminotransferase fold. The N-terminal arm is coloured in black. The large PLP-binding domains from the A and B subunits are coloured in cyan and green, respectively. The smaller C-terminal domains are coloured in purple and yellow, respectively. The two insertions 150–164 and 212–217 that are found only in Spr1654 are coloured in blue and red, respectively. (*b*) Interface and active sites within the Spr1654 homodimer. The surface and the homodimer interface within one of the two subunits are coloured in cyan and wheat, respectively. The cartoon representation of the other subunit is coloured in rainbow, with the disordered region coloured in grey. The active site is indicated in one of the two subunits.

The crystal structure of the Spr1654 monomer adopts a type I aminotransferase fold (figure 3*a*). Each Spr1654 subunit can be divided into an N-terminal arm (residues 1–14), a large PLP-binding domain comprising residues 15–276 and a smaller C-terminal domain comprising residues 277–404. The large PLP-binding domain of Spr1654 contains a central seven-stranded mixed β-sheet with the order β3–β2–β4–β5–β8–β9–β1. Strand 9 is antiparallel to all the other β-strands (figures 2 and 3*a*). Ten α-helices are flanking the central β-sheet from both sides. The smaller C-terminal domain starts after a kink in the helix α10 at residue Pro276 and contains a three-stranded antiparallel β-sheet comprising strands 12, 15 and 16, as well as five helices that encircle the β-sheet from three different sides (figure 3*a*). Two large and deep cavities are formed at the homodimer interface, where the two presumptive active sites are located (figure 3*b*). The inter-subunit interactions within each homodimer extensively involve the four α helical segments α1 (including flanking loops), α4, α11 (with loops) and α12, as well as the β-strand β11 and several loops (figures 2 and 3*b*). Interactions at the dimer interface are stabilized by 21 hydrogen bonds/salt bridges combined with a large amount of hydrophobic interactions.

Importantly, Spr1654 has a unique feature compared with other aminotransferases: it is longer with two insertions comprising the stretches of residues 150–164 covering the helix α7, and residues 212–217 which form the loop that links the strand β9 and the helix α8 (figures 2 and 3*a*). Although these regions are separated in the amino acid sequence of Spr1654, they are localized very close to each other in the crystal structure, covering the shallow depression on the surface, present on other SAT (figure 3*a*). They are also located relatively close to the entrance of the active site (figure 3). The possible functional role of these unique insertions will be discussed later in the section related to substrate binding.

Using the Dali server [26], we compared the three-dimensional structure of Spr1654 with all known structures within the PDB. The obtained results coincide very well with the sequence blast search (figure 2). The best hits indicated the sugar aminotransferases ArnB (PDB code: 4OCA and 1MDO [20,27]), PseC (PDB code: 2FNU [22]) and GDP-perosamine synthase (PDB code: 3DR7 [21]) as the highest structural homologues, with *Z*-scores of 48.3, 49.1 and 49.5, respectively. The conserved substrate-binding pockets and active sites found in the crystal structure of Spr1654 further support its aminotransferase role in biosynthesis of TAs (figures 2 and 3*b*).

### 2.3. PLP binding in Spr1654 occurs via residues conserved among aminotransferases

**Figure 4.**
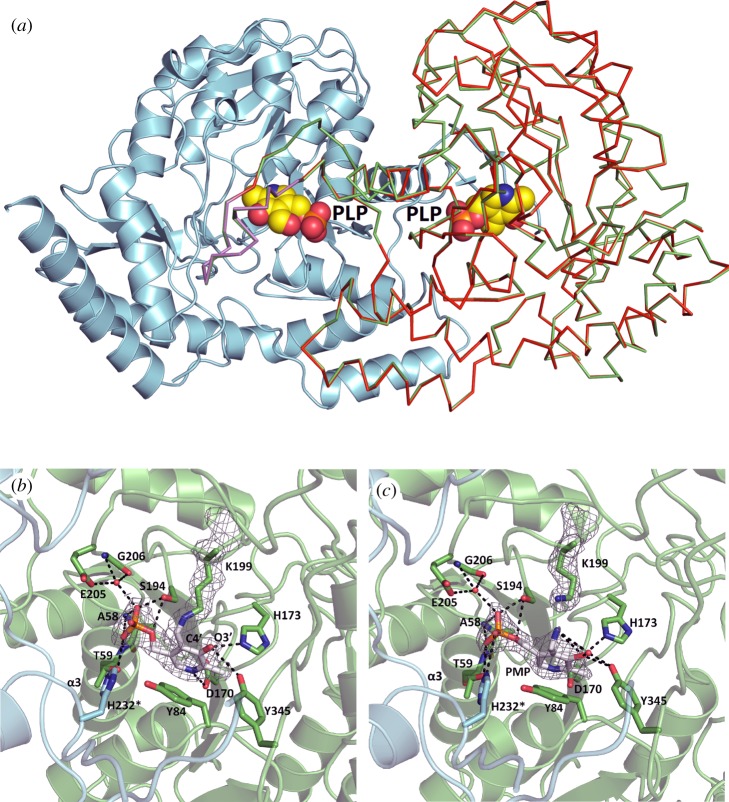
Cofactor-binding site in Spr1654 dimer. (*a*) PLP binds in the two deep and wide cavities. The two subunits bound to PLP are coloured in cyan and green. The apo-form of one subunit of Spr1654, coloured in red, is superposed to one PLP-bound subunit, demonstrating that no conformational change occurs upon PLP binding. Both PLP cofactors are displayed in space-filling representation. The disordered region comprising residues 235–248 are coloured in blue and violet in the Spr1654/PLP complex and apo-Spr1654, respectively. (*b*) The bound PLP forms a covalent bond with the catalytic residue Lys199. The cofactor and Spr1654 residues, important for binding and catalysis, are displayed as sticks. Hydrogen bonds and salt bridges are indicated with black dashed lines. The bridging water is displayed as a red sphere. The electron density map is displayed at 1*σ* contour level. (*c*) The crystal structure of Spr1654 in complex with PMP reveals no conformational change, except for the tip of the side chain of residue Lys199. The electron density map is displayed at 1*σ* contour level.

In the crystal structure of the Spr1654/PLP complex, the PLP-binding sites are located at the bottom of deep and wide cavities formed at the interfaces between the subunits (figure 3*b*). The contribution from both subunits of the homodimer is essential for the formation of the active sites. The aromatic ring of PLP sits at the deep end of each cavity (figure 4*a*). Clear electron density confirms the formation of a covalent bond between PLP and residue Lys199 in Spr1654 (figure 4*b*). Although the quality of the electron density is reduced in subunits C and D, the conformation of Lys199 confirms the presence of the internal aldimine in all subunits.

The N1 atom of PLP is positioned at 2.8 and 3.2 Å from the OD1 and OD2 atoms of the conserved residue Asp170 (figures 2 and 4*b*). These interactions enhance the electron sink nature of PLP and maintain the cofactor in a protonated state, which is a typical feature of the aspartate aminotransferase (AAT) type 1 enzyme family [28]. The deprotonated O3′ of PLP is at a hydrogen-bond-forming distance from the side chain of residue His173, which preserves the negative charge of O3′, and stabilizes the protonated internal aldimine of Spr1654 (figure 4*b*). The side chain of residue Tyr84 in Spr1654 forms van der Waals contact with the pyridine ring of PLP. These two aromatic rings are not parallel to each other, forming instead an angle of approximately 60° between their planes, such that atom CD2 in Tyr84 becomes the closest atom to the C3 and C4 atoms in PLP (figure 4*b*). These interactions are conserved in all known sugar aminotransferases even though the aromatic residues are not conserved. Indeed, ArnB has a tryptophane while PseC and other sugar aminotransferases from *E. coli* comprise a phenylalanine at this position (figure 2). It has been suggested that this residue, corresponding to Tyr84 in Spr1654, dictates the correct position of the substrate, localizing it close to the C4′ atom of PLP [27]. The negative charge of the phosphate group of the cofactor is neutralized by the dipole of the helix α3 (figures 2 and 4*b*). In addition, the phosphate group forms two hydrogen bonds with the side chains of residues Ser194 and Thr59 in Spr1654, as well as water-mediated hydrogen bonds with the conserved residues Glu205 and Gly206 (figure 4*b*).

In the crystal structure of the Spr1654/PMP complex, the phosphate group serves as the main anchor, keeping PMP in the active site. Disruption of the covalent bond between Lys199 and PLP pushes the NZ atom away from the cofactor (figure 4*c*) without any distortion of the position of any other residue. PMP moves by 0.5–0.6 Å in the opposite direction, away from the bottom of the cleft, which results in a small perturbation of the hydrogen bond network, although the main interactions remain intact. Consistent with the comparison between the apo- and ligand-bound forms of other SATs, nothing seems to happen with Spr1654 upon PLP/PMP dissociation. Indeed, the crystal structure of the apo-form of Spr1654 indicates that water molecules fill the cofactor-binding cavity, without any alteration of the conformation of any residue (data not shown).

### 2.4. l-glutamate serves as the optimal amino donor for Spr1654

Aminotransferases usually catalyse the transfer of an amino group between an amino acid and a specific oxoacid substrate. As in all transamination reactions, sugar aminotransferases transfer an amino group from an amino acid donor to an NDP-activated keto sugar. For most SATs, the amino donor is either l-glutamate or l-glutamine [25], but l-aspartate may also be used in a few cases [29]. As for other aminotransferases, the enzymatic reaction of SATs is supposed to follow a well-established mechanism, divided into two half-reactions [25,30]. In the first step, and in the absence of substrate, the enzyme reacts with the amino donor to yield enzyme–PMP and a novel oxoacid [31]. In the second step, the enzyme–PMP complex binds to a keto sugar nucleotide acceptor. It has been previously proposed that UDP-4-keto-6-deoxy-d-GlcNAc forms the product UDP-AAT-Gal in order to regenerate Spr1654–PLP (figure 1*b*) [5].

**Figure 5.**
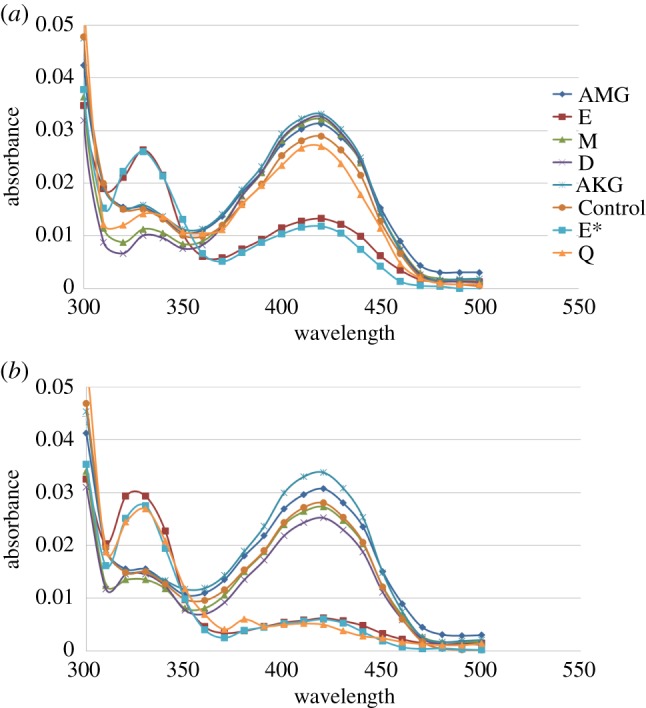
Qualitative transamination half-reaction assay demonstrates that l-glutamate serves as an amino donor substrate for Spr1654. Spectra of the enzyme (*a*) before and (*b*) after 1 h incubation were collected between 300 and 500 nm. The appearance of a 330 nm peak indicates the conversion of the enzyme-bound PLP to PMP. AMG, 2-methylglutamic acid; E, l-glutamate; M, l-methionine; D, l-aspartate; AKG, α-ketoglutarate; Q, l-glutamine. Assays with l-glutamine (Q) were performed separately using l-glutamate as control (E*).

The natural amino donor for Spr1654 has hitherto remained unknown. We therefore performed a qualitative transamination half-reaction assay in order to validate the optimal substrate for Spr1654 (figure 5). Both PLP- and PMP-formed enzymes give different absorbance spectrum profiles, with the appearance of a 330 nm absorbance peak (characteristic of PMP formation) indicating a positive amino donor [32]. We analysed several amino acids including 2-methylglutamic acid (AMG), l-glutamate (E), l-glutamine (Q), l-methionine (M), l-aspartate (D) and α-ketoglutarate (AKG), based on a qualitative transamination half-reaction study (figure 5). Upon the addition of l-glutamate, absorbance changes in the visible region occurred immediately, resulting in a significant decrease of the 420 nm PLP absorbance and the concomitant appearance of a 330 nm PMP absorbance peak (figure 5*a*). Interestingly, for l-glutamine, although we could not see the significant absorbance differences within the first few minutes (figure 5*a*), the reaction occurred following incubation for a significantly longer time period (at least 1 h) (figure 5*b*). By contrast, no obvious spectra changes at 330 and 420 nm occurred for l-methionine and l-aspartate, even after 1 h incubation (figure 5*b*). As expected, the PLP peak was slightly increased for AKG by running the first half-reaction in reverse, while for the glutamate analogue AMG, the peak absorbance remained unchanged. Thus, our results demonstrate that Spr1654 is able to deaminate l-glutamate in the first half-reaction of transamination with high efficiency and indicate that l-glutamate is the preferred amino donor that is converted to α-ketoglutarate in the first half-reaction.

**Figure 6.**
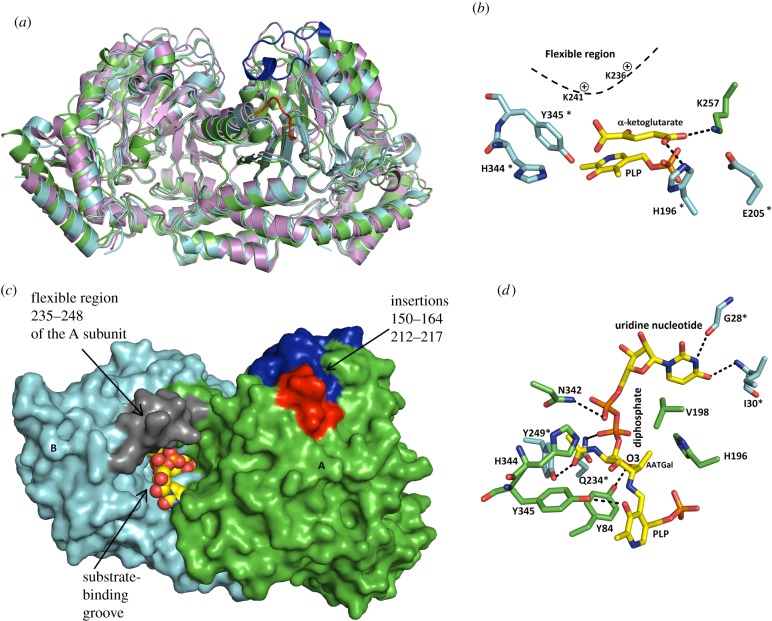
Molecular models of Spr1654 in complex with substrates indicate that no conformational changes are required for ligand binding. (*a*) Superposition of Spr1654 on ArnB from *S. typhimurium* (PDB code: 4OCA) and PseC from *H. pylori* (PDB code: 2FNU). Spr1654, ArnB and PseC are coloured in green, cyan and violet, respectively. The two major insertions 150–164 and 212–217, found only in Spr1654, are coloured in blue and red, respectively. (*b*) Docking of α-ketoglutarate in the active site of Spr1654 indicates possible key residues that interact with the substrate. The flexible region comprising residues 235–248 is in dashed line and the two positive charged lysine residues are indicated. (*c*) The substrate-binding site of Spr1654 can accommodate UDP-AATGal without any conformation alteration. Substrate atoms and PLP are displayed as balls. The flexible region is coloured in grey, while the unique insertions are in blue and red, respectively. (*d*) The UDP-AATGal binds snuggly within the active site of Spr1654. Substrate atoms and Spr1654 residues possibly important for binding are shown as sticks, coloured in cyan and green for subunits A and B, respectively. Hydrogen bonds are indicated with black dashed lines.

The crystal structure of Spr1654/PLP in complex in the presence of l-glutamate did not reveal any electron density for l-glutamate (data not shown), indicating that the enzymatic reaction takes place in the obtained crystals and that the products were not trapped in the binding site. The crystal structure of ArnB has been previously determined in complex with the product α-ketoglutarate in the active site [27]. In most of the hitherto studied aminotransferases with a type I fold, such as the AAT, binding of the substrate results in a large interdomain movement [33]. In contrast to AAT, ligand binding to SAT does not induce any significant conformational changes, as previously shown for ArnB [20,27] and PseC [22]. The crystal structure of *S. pneumoniae* Spr1654 was superposed on the previously determined structures of aminotransferase PseC (PDB code: 2FNU [22]) and ArnB (PDB code: 1MDX or 4OCA [20,27]). The intrinsic rigidity of the active sites within these aminotransferase (figure 6*a*) allowed us to create a molecular model of the enzyme–substrate complexes. Following superposition of the two crystal structures of Spr1654 and ArnB (PDB ID: 1MDX), the α-ketoglutarate could be docked in the active site of Spr1654 without any distortion of the protein (figure 6*b*). Analysis of the molecular model of α-ketoglutarate within the active site of Spr1654 allowed us to identify residues possibly responsible for l-glutamate binding and specificity. The α-ketoglutarate is located between a large ensemble of polar residues from both subunits (figure 6*b*). Furthermore, it is positioned close to the helix α9 that is disordered in subunits B and D of Spr1654 (figures 3*a* and 6*b*), similarly to the PLP/PMP forms of ArnB [27]. The addition of α-ketoglutarate or co-crystallization with PLP-UDP-Ara4N ordered this flexible region within ArnB [20,27].

Usually, AATs bind dicarboxylic substrates, such as α-ketoglutarate, between two arginine residues. Fork-like salt bridges formed between the substrate carboxylic groups and the guanidinium groups of these two arginines are essential for the enzymatic reaction [34]. These two fundamental arginine residues are not present in Spr1654 or in other sugar aminotransferases. Indeed, SATs make use of a different motif for binding to amino donors. The γ-carboxyl groups of either α-ketoglutarate or l-glutamate interact with the side chains of Lys257 (Lys241 in ArnB or asparagine residues in 2FNU and 3DR7) and His196 (His185 in ArnB, phenylalanine in 3DR7) (figures 2 and 6*b*). Residue Glu205, conserved among all SATs, participates in the substrate specificity of Spr1654, coordinating the orientation of His196 and Lys254. In contrast to AATs, with two arginine residues that restrict the amino donor selection to only dicarboxylic substrates, the binding motif in Spr1654 allows for both l-glutamate and l-glutamine to be used as amino donors.

According to our molecular model, the other end of α-ketoglutarate most probably binds to residues localized on the flexible region of Spr1654 (residues 235–248, figure 6*b*,*c*). The sequence of the flexible region, which also participates in the binding of the second substrate, is not conserved among SATs (figure 2). While ArnB makes use of Arg229 to bind to the α-carboxyl of α-ketoglutarate [27], no such basic residue is present at this position in any other SAT. However, this region contains one or two positively charged residues in all SATs (figure 2), such as Lys236 and/or Lys241 in Spr1654, which both could bind to the α-carboxyl group of α-ketoglutarate (figures 2 and 6*c*). It therefore remains possible that l-glutamate binds differently than proposed by our molecular model, or that l-glutamate binding induces conformational changes within the flexible stretch of residues 235–248 (figure 6*c*). Nevertheless, it is clear that the distance between the two carboxyl groups of the amino donor is essential for substrate selectivity, explaining why methionine or aspartate displays no activity in the qualitative transamination half-reaction assay (figures 5 and 6*b*). Furthermore, compared with l-glutamate, l-glutamine cannot form sufficiently strong interactions with Lys257, resulting in low enzymatic efficiency. Altogether, our study indicates that l-glutamate serves as the optimal donor for the transaminase activity of Spr1654.

### 2.5. A molecular model of the external aldimine of PLP-UDP-AATGal Spr1654 indicates the importance of several conserved residues for substrate binding

As mentioned above, ligand binding does not result in any interdomain movement in SATs. Instead, it results in the ordering of the flexible region, corresponding to the stretch of residues 235–248 in Spr1654, which could be essential for the enzymatic reaction. All our co-crystallization efforts have been hampered by the lack of a commercial source for the possible biological substrate UDP-4-keto-6-deoxy-d-GlcNAc and the corresponding product UDP-AATGal [5]. We therefore created a molecular model of Spr1654 in complex with the UDP sugar by superposing the crystal structure of the Spr1654/PLP complex on the previously determined structures of PseC bound to PMP-UDP-4-amino-4, 6-dideoxy-l-AltNAc (PMP-UDP-l-AltNAc) [22] and ArnB bound to UDP- 4-amino-4-deoxy-l-Arabinose (UDP- Ara4N) [20] (figure 6). UDP-AATGal fits perfectly within the binding groove of Spr1654, without any requirement for the alteration of the conformation of either substrate or any Spr1654 residues, including the ordered 235–248 region (figure 6*c*). It binds within the groove leading to the cavity that contains PLP and is stabilized by interactions with several residues from both monomers (figure 6*d*). The position of PMP remains unmodified. The galactose ring stacks between the phenyl ring of residues Tyr84 and Val198. The two conserved histidine residues His196 and His344 flank the galactose ring in the equatorial plane (figure 6*d*).

We also find it interesting that all sugar aminotransferases have an unusual non-proline *cis*-bond formed between the conserved residues His344-Tyr/Phe345. Both these residues line the substrate-binding cavity close to AATGal in the substrate. It is possible that this *cis*-bond provides the correct orientation of these residues (figure 6*d*). The *N*-acetyl group of the sugar substrate is located in a large side cavity formed by residues Gln234*, Tyr345 and Tyr249*, leading towards the protein surface, and could form hydrogen bonds with Tyr249* from the disordered region of subunit B (figure 6*c*,*d*). Moreover, residue Tyr84 in Spr1654 (Phe84 in PseC) could be important for fitting the *N*-acetyl group of the substrate within the active site. By contrast, the larger residue Trp89 in ArnB allows only for a sugar substrate without *N*-acetyl group (electronic supplementary material, figure S2). The methyl group of the galactose ring of the substrate also fits perfectly in a nearby pocket in which it is surrounded by the conserved residues His196 (His180 in PesC and His185 in ArnB) and Val198 (Ile182 in PesC and Ile187 in ArnB) (figures 2 and 6*d*). The molecular model also indicates that while the galactose O3 atom forms a hydrogen bond with the hydroxyl of residue Tyr84, the hydrogen bond formed between the O3 atom of PLP and Tyr345 remains the same as in the crystal structure of Spr1654 in complex with PLP (figure 6*d*).

The diphosphate oxygens of UDP form hydrogen bonds with the side chains of residues Gln234* and Asn342 (figure 6*d*). The negative charge of the diphosphate moiety can be neutralized in Spr1654 by the two positively charged residues Lys236 and Lys241 both from the highly flexible 235–248 region (figure 6*c*). Although these two lysine residues are positioned far away from the substrate diphosphate (7–10 Å), a conformational change in this mobile region could occur during substrate binding and bring these two lysine residues closer to the substrate.

The uridine nucleotide binds with its uracil base in a pocket formed at the dimer interface, forming hydrogen bonds with the second monomer. Both its O4 carbonyl and N3 atom form hydrogen bonds with residue Ile30* and the carbonyl of residue Gly28*, respectively, both localized on subunit A (figure 6*d*). The ribose moiety does not form any hydrogen bond and interacts with Spr1654 only through several van der Waals contacts.

It has been previously suggested that in some sugar biosynthetic pathways, the 4-keto-6-deoxy intermediates produced by nucleotide-sugar dehydratases are unstable [35,36]. In *S. pneumoniae*, Spr0092 presumably makes use of UDP-GlcNAc to form UDP-4-keto-6-deoxy-d-GlcNAc, in order for Spr1654 to thereafter generate UDP-AATGal through the transaminase reaction (figure 1*b*). However, in our hands, no corresponding products were detected using several different soluble version of the Spr0092 dehydratase domain (data not shown). Thus, it could be possible that Spr1654 forms a multiprotein complex with the transmembrane Spr0092 for the efficient transport of the intermediates between the two active sites. Two unique insertions in the sequence of Spr1654, corresponding to residues 150–164 and 212–217 that distinguished Spr1654 from Gram-negative bacteria-derived SATs, are localized close to the entrance of the substrate-binding groove (figure 6*b*) and could be important for the formation of such a multiprotein complex.

### 2.6. Biological implications

*Streptococcus pneumoniae* remains one of the leading bacterial causes of mortality worldwide, and the development of new drugs is acutely needed especially considering the fast emergence of drug-resistant strains. Spr1654 is involved in the early steps of TA precursor synthesis, where the first amino sugar UDP-AATGal is suggested to be synthesized by this aminotransferase, followed by several TA biosynthesis pathway steps. Here, we have determined the crystal structures of Spr1654 in the apo-form as well as in complex with PLP and PMP. Our amino donor assays demonstrate that l-glutamate is the optimal amino donor for Spr1654 and molecular modelling indicates that UDP-AATGal can be accommodated within the active site of Spr1654. Altogether, our results demonstrate the aminotransferase activity of Spr1654 and support its previously proposed role in the TA synthetic pathway. Whether Spr1654 could use the proposed substrate UDP-4-keto-6-deoxy-d-GlcNAc to form UDP-AATGal remains to be determined. We believe that our structural results might also serve as a starting point for structure-based antimicrobial compound design.

## 3. Material and methods

### 3.1. Cloning

All protein constructs were cloned into pET-23b expression vector (Novagen) using the ligation-independent Fast Cloning method (LIC). The coding sequence for the full-length spr1654 (GenBank: ABJ54416.1) [37] was synthesized according to *S. pneumoniae* TIGR4 chromosomal DNA and used as a template for PCR to generate the expression construct with a C-terminal poly-histidine (HHHHHH). The ligation mixture was transformed into *E. coli* competent cell TOP10 and a positive clone was selected. All coding sequences of the protein-expression constructs were confirmed by DNA sequencing.

### 3.2. Expression and purification of Spr1654

The resulting plasmid pET-23b-Spr1654 was transformed into T7 express competent cells for overexpression of full-length native Spr1654. Cells were cultivated at 37°C in LB medium containing 100 µg ml^−1^ ampicillin until the optical density reached 0.8. The culture was cooled down for 30 min and overexpression was induced by adding isopropyl-1-thio-β-d-galactopyranoside to a final concentration of 0.5 mM and expressed overnight at 16°C with shaking at 225 r.p.m. All the purification steps were carried out at 4°C. Poly-histidine-tagged Spr1654 was purified by a nickel–nitrilotriacetic acid (Ni-NTA) Superflow column (Qiagen) and a Superdex200 Increase 10/300 GL gel filtration column (GE Healthcare) pre-equilibrated with buffer containing 20 mM Tris–HCl (pH 7.5) and 200 mM NaCl. Fractions containing highly purified Spr1654 as judged by SDS–PAGE and were pooled and concentrated with 30 K Vivaspin turbo centrifugal concentrator (MWCO). Purity of his-tagged Spr1654 was assessed by SDS–PAGE to be at least 99%.

### 3.3. Crystallization of apo-Spr1654, Spr1654/PLP and Spr1654/PMP

Spr1654 protein was concentrated to 20 mg ml^−1^ in 20 mM Tris–HCl (pH 7.5), 200 mM NaCl using a 30 kDa cut-off Vivaspin concentrator (Vivaspin). For Spr1654/PLP complex, 1 mM PLP was added into 1.5 mg ml^−1^ Spr1654, incubating at 20°C for 2 h, and the mixture was concentrated to 17.8 mg ml^−1^, by which excessive PLP was removed. For the PMP-bound enzyme, additional l-glutamate was added into Spr1654/PLP complex before crystallization. Crystallization trail was performed using Crystal Screen HT, Index HT and PEG/Ion HT (Hampton Research), JSCG+ (Qiagen) and Morpheus (Molecular Dimensions). All crystallization experiments were carried out with sitting drop in 96-well plates at 20°C. The volume ratio of protein to crystallization reservoir solution varied between 1 : 1, 1 : 2 and 2 : 1. Crystals of apo-Spr1654, Spr1654/PMP and Spr1654/PLP were obtained under several conditions. The crystals grown in reservoir solution of 0.2 M sodium acetate trihydrate, 0.1 M sodium cacodylate trihydrate (pH 6.5) and 30% (w/v) polyethylene glycol 8000 gave the best diffraction.

### 3.4. Data collection and processing

Crystals were soaked in a cryo-protectant solution containing 20% ethylene glycol before flash freezing in liquid nitrogen. Data collection was performed under cryogenic conditions (100 K) at beam line ID30B (ESRF, Grenoble, France) and beam line 14.1 (Bessy, Berlin, Germany). Data were processed with MOSFLM [38] and AIMLESS [39] of CCP4 suite. Data collection statistics are provided in table 1.

### 3.5. Crystal structure determination and refinement

The crystal structures were determined by molecular replacement in Phaser [40] using the crystal structure of UDP-4-amino-4-deoxy-l-arabinose-oxoglutarate aminotransferase from *Burkholderia cenocepacia* (PDB code: 4LC3) as search model. Phaser found the solution with four molecules in the asymmetric unit. Five per cent of the total amount of reflections were set aside for monitoring refinement by *R*_free_. Refinement of the crystal structure was performed using REFMAC [41] and Phenix [42]. A clearly interpretable electron density was observed, and the PLP could be unambiguously modelled in all the four molecules in the asymmetric unit. Water molecules were added using COOT [43] and their position was inspected manually. The stereochemistry of the final models was verified with COOT. The final refinement parameters are presented in table 1. Figures were created using PyMOL 1.8 (Schrödinger, LLC).

### 3.6. Spectroscopic measurements

Absorption spectra were made with a SpectraMax Plus384 microplate reader. The enzyme solution was centrifuged at 10 000 r.p.m. to reduce light scattering from a small amount of precipitate. Reaction progress was followed by monitoring the absorbance maxima of the PMP or PLP cofactor forms at 330 nm or 420 nm, respectively. The reaction was carried out in 50 mM MES (pH 6.6), 200 mM NaCl containing 0.9 mg ml^−1^ Spr1654/PLP and different amino acids (0.5 mM l-glutamate, l-methionine, l-aspartate or α-ketoglutarate) at 25°C. The total reaction system was 100 µl. The absorbance of the sample was measured against a blank the same components except enzyme. The absorbance was measured at 300–500 nm at a stepwise 10.

## Supplementary Material

Figure S1

## Supplementary Material

Figure S2

## Data accessibility

All data are accessible within the manuscript or in the electronic supplementary material. Coordinates and structure factors have been deposited in the PDB under accession codes 6EWJ, 6EWQ and 6EWR.
