# Supplementary material for: Structural and functional studies of Spr1654: an essential aminotransferase in teichoic acid biosynthesis in Streptococcus pneumoniae
Source: Open Biol. 2018 Apr 18;8(4):170248. doi: 10.1098/rsob.170248 (PMC5936713; doi:10.1098/rsob.170248)
Supplement: Figure S1 [file rsob170248supp1.pdf]

Supplementary Figure

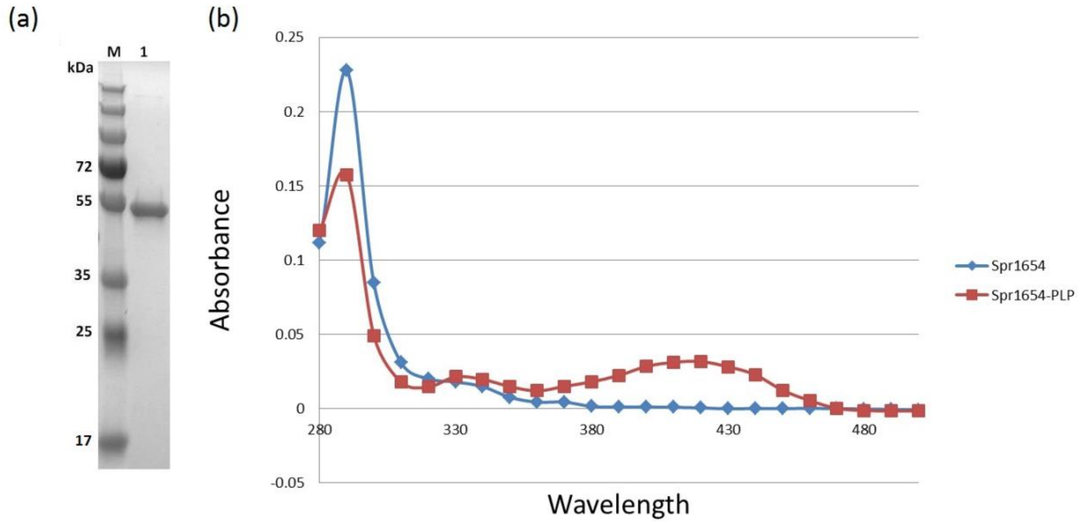

Figure S1. Absorption spectrum of Spr1654 and Spr1654-PLP. (a) Analysis of recombinant Spr1654-his (MW 47.7 kDa) by SDS-gel electrophoresis. The protein was separated by SDS-12% PAGE, followed by staining with Coomassie blue. Lane 1, Spr1654-his; M, molecular mass marker. (b) The absorption spectra of Spr1654 with (red line) and without PLP (blue line) were collected between 300 and 500 nm. The appearance of a 420nm peak indicates PLP binding to Spr1654.
