# Supplementary material for: Structural and functional studies of Spr1654: an essential aminotransferase in teichoic acid biosynthesis in Streptococcus pneumoniae
Source: Open Biol. 2018 Apr 18;8(4):170248. doi: 10.1098/rsob.170248 (PMC5936713; doi:10.1098/rsob.170248)
Supplement: Figure S2 [file rsob170248supp2.pdf]

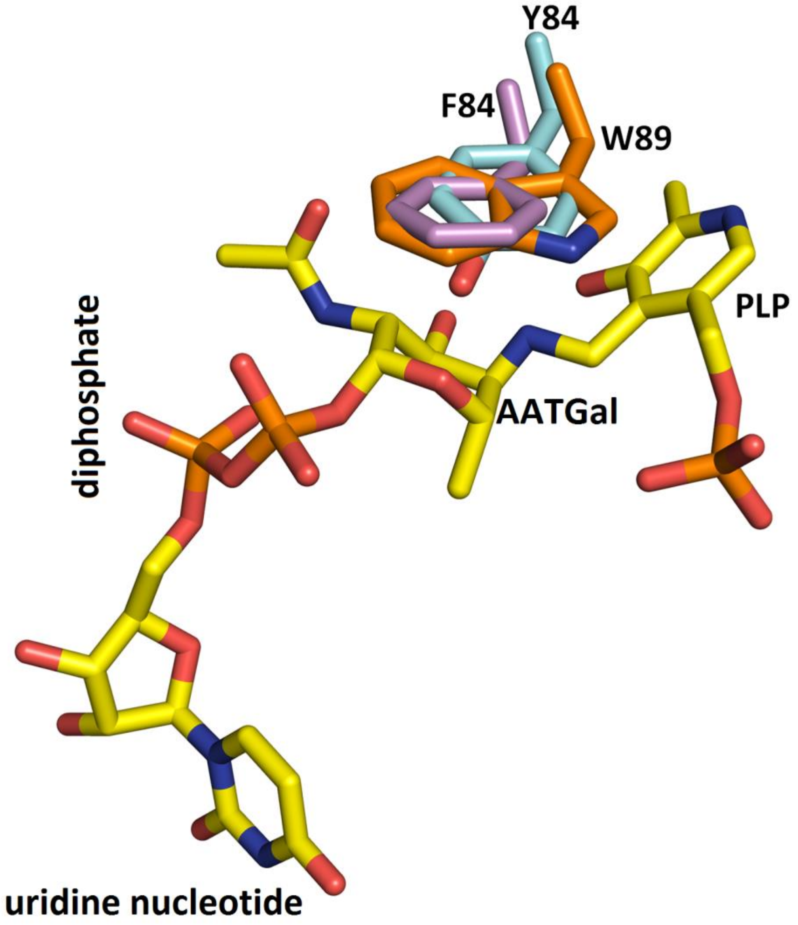


**Figure S2.** Residue Tyr84 in Spr1654 allows enough space for accommodating the N-acetyl group of the sugar substrate. Residues Y84 in Spr1654, W89 in ArnB and F84 in PseC are colored in cyan, orange and violet, respectively. PseC binds to PMP-UDP-4-amino-4, 6-dideoxy-L-AltNAc (PMP-UDP-L-AltNAc) and ArnB binds to UDP- 4-amino-4-deoxy-L-Arabinose (UDP- Ara4N).
